# Mining Beneficial Genes for Salt Tolerance From a Core Collection of Rice Landraces at the Seedling Stage Through Genome-Wide Association Mapping

**DOI:** 10.3389/fpls.2022.847863

**Published:** 2022-04-26

**Authors:** Xiaoliang Wang, Jinquan Li, Jian Sun, Shuang Gu, Jingbo Wang, Chang Su, Yueting Li, Dianrong Ma, Minghui Zhao, Wenfu Chen

**Affiliations:** ^1^Rice Research Institute, Shenyang Agricultural University, Shenyang, China; ^2^State Key Laboratory for Conservation and Utilization of Subtropical Agro-Bioresources, South China Agricultural University, Guangzhou, China; ^3^Strube Research GmbH & Co. KG, Söllingen, Germany

**Keywords:** rice, salinity tolerance, candidate gene, GWAS, QTL

## Abstract

Rice is a salt-sensitive plant. High concentration of salt will hinder the absorption of water and nutrients and ultimately affect the yield. In this study, eight seedling-stage salt-related traits within a core collection of rice landraces were evaluated under salinity stress (100 mM NaCl) and normal conditions in a growth chamber. Genome-wide association study (GWAS) was performed with the genotypic data including 2,487,353 single-nucleotide polymorphisms (SNPs) detected in the core collection. A total of 65 QTLs significantly associated with salt tolerance (ST) were identified by GWAS. Among them, a co-localization QTL *qTL4* associated with the SKC, RN/K, and SNC on chromosome 6, which explained 14.38–17.94% of phenotypic variation, was selected for further analysis. According to haplotype analysis, qRT-PCR analysis, and sequence alignment, it was finally determined that 4 candidate genes (*LOC_Os06g47720*, *LOC_Os06g47820*, *LOC_Os06g47850*, *LOC_Os06g47970*) were related to ST. The results provide useful candidate genes for marker assisted selection for ST in the rice molecular breeding programs.

## Introduction

Soil salinization is one of the important abiotic stresses that limits agricultural food production, because it reduces crop yields and restricts land use. With the modernization of industry and the continuous deterioration of the ecological environment, the area of arable land is gradually decreasing, while the area of soil salinization continues to increase because of unreasonable irrigation to the farmland (Qadir et al., 2014). It is estimated that about 950 million hectares of arable land in the world, including 250 million hectares of irrigated land, are affected by salinization (Solis et al., 2020). Rice (*Oryza sativa*) is a salt-sensitive plant, and salt stress seriously affects its growth, development, and yield (Wang et al., 2018a). Therefore, it is of great significance to mine and utilize the novel salt-tolerant (ST) genes in rice and breed ST varieties (Li et al., 2014).

A large number of studies have shown that ST is a complex trait controlled by quantitative trait loci (QTL; Roy et al., 2011). In recent years, with the development of modern science and technology, sequencing technology has become more and more mature. A lot of researches have been done on QTL mapping of ST in rice, and many QTLs related to ST have been identified. Using the F_2_ population derived from the parent Dianjingyou1 as the recipient parent and SR86 as the donor parent, a huge segregation analysis library was constructed and a QTL named *qst1.1* related to ST was identified on chromosome 1, which can explain 62.6% of the phenotypic variation (Wu et al., 2020). Using the RIL population, 38 QTLs related to ST were identified and a new QTL affecting stem length was found on chromosome 7, which could explain 6.8% of the phenotypic variation (Jahan et al., 2020). Takehisa et al. (2004) identified 12 QTLs for stem length under salt stress on chromosomes 1, 3, and 7, explaining 12–30% of the phenotypic variation. Wang et al. (2012) used the IR26/Jucaiqing recombinant inbred line (RIL) population and found 16 QTLs related to the swelling rate and germination rate under salt stress. Prasad et al. (2000) used the DH population and identified a QTL for seed root length under salt stress on chromosome 6, which explained 18.9% of the phenotypic variation. Zeng et al. (2021) used the BC_1_F_2_ population and performed QTL mapping for the seed germination rate (GR) and germination index (GI). A total of 13 QTLs were identified, which explained 7.32–24.39% of the phenotypic variation.

GWAS can be used to perform association analysis on the genetic variation of complex traits at the genome-wide level (Hirschhorn and Daly, 2005). It is an effective method for in-depth understanding of the genetic structure of complex traits of crops. With GWAS, several researchers have identified many QTLs and candidate genes related to ST traits. Yuan et al. (2020) used 664 rice varieties and different statistical models to conduct GWAS for ST, and a total of 21 QTLs were identified. Using 208 rice varieties from a core collection, Naveed et al. (2018) identified 20 quantitative trait nucleotides (QTNs) for one salt related trait through GWAS, including 6 QTNs affecting ST at the germination stage and 14 QTNs at the seedling stage, and identified 22 candidate genes. Using 708 rice varieties, Liu et al. (2019) identified 7 candidate genes through GWAS, which were significantly associated with grain yield and its related traits under saline stress conditions. Zhang et al. (2017) used GWAS to identify salt-tolerant loci and favorable alleles for iron and zinc resistance and detected 60 salt-tolerant loci as well as 22 candidate genes in 10 important QTLs regions. An et al. (2020) used a core collection which consisted of 181 varieties and detected 17 loci significantly associated with dry weight ratio (DWR) for ST through GWAS.

The seedling stage is the key stage for ST in rice, and it is highly sensitive to salt stress (Nayyeripasand et al., 2021). High concentration of salt will hinder the absorption of water and nutrients in the rice seedling stage, inhibit the growth of seedlings, and ultimately reduce rice yield (Ruan et al., 2011). To a certain extent, it can be used as a reference for ST during the whole growth period. The ST identification at the seedling stage is easy to operate with short cycle and high efficiency and can be widely used in screening germplasm resources for ST and breeding selection (Walia et al., 2005).

Abundant germplasm resources for ST are available in the Asian cultivated rice, especially in rice landraces. As early as in 1920–1964, a total of 7,128 accessions of rice landraces had been collected by Prof. Ying Ting, which was named as Ting’s rice collection (Li et al., 2011). They were from all over China as well as from some main rice cultivation countries. Based on 48 phenotypic data, Li et al. (2011) have constructed a rice core collection consisting of 150 accessions. The large variation within the core collection provides an important gene pool of genetic diversity and beneficial genes for rice breeding. Therefore, it is worth to perform GWAS with such a core collection for ST in rice. Population structure analysis for the core collection indicated that there existed two subgroups mainly corresponding to *indica* and *japonica* subspecies and the linkage disequilibrium (LD) decay distance was about 200–500 kb (Zhang et al., 2011; Li and Zhang, 2012; Zhao et al., 2018). Using this core collection, several researches have been performed to identify resistant QTLs for aluminum (Al) tolerance and cold tolerance. With the mixed linear model, GWAS identified a total of 30 QTLs for Al tolerant traits which explained 7.73 to 13.30% of the phenotypic variation (Zhao et al., 2018). A total of 26 QTLs were found to be significantly associated with cold tolerance, which explained 26–33% of the phenotypic variation (Song et al., 2018). These results indicated that these landraces are importance sources for stress tolerance in rice and the mapping results could provide important information to breed stress tolerant rice cultivars through marker-assisted selection. However, to our knowledge, no previous research was performed for ST at the seedling stage with the core collection. Moreover, no previous research was performed to map QTLs for ST and further identify candidate genes with the core collection. Therefore, the objectives for this study were: (1) to screen the performance of ST in the in the core collection; (2) to map the QTLs for ST through GWAS; and (3) to identify some candidate genes for ST in rice for better understanding the genetic basis of ST at the seedling stage in rice and providing new genetic resources for improvement of ST in rice cultivars.

## Materials and Methods

### Plant Materials

The Ting’s rice core collection, i.e., a total of 150 accessions of rice landraces, was used to screen their salt tolerance (Supplementary Table S9). These landraces were mainly collected from 20 different provinces in China as well as from North Korea, Japan, Philippines, Brazil, Celebes, Java, Oceania, and Vietnam. These regions are distributed across the north latitude 55° to south latitude 10° and including regions with temperate, tropical, and subtropical climate. The core collection was constructed from 150 accessions of 2,262 based on a strategy of stepwise clustering and preferred sampling on adjusted Euclidean distances and weighted pair-group average method using integrated qualitative and quantitative traits (Li et al., 2007). Of the 150 landraces, 32 were classified as *japonica* rice (24 were typical *japonica* rice, and 8 were *japonica*-clined rice), and 118 were classified as *indica* rice (16 were *indica*-clined rice, and 102 were typical *indica* rice), according to Cheng’s index criterion (Li et al., 2011).

### ST Evaluation at the Seedling Stage

The seeds of the 150 landraces were placed in an oven at 50°C for 48 h to break dormancy. From each landrace, 40 seeds with uniform size and full rice grains were selected, and then the seeds were soaked in 75% alcohol for 15–20 min for disinfection treatment. Then, the seeds were rinsed with sterile water for three times. The rinsed seeds were put in a net bag, soaked in distilled water, and placed in a thermostat at 30°C for 48 h to incubate germination. Then, the seeds were transferred to 96-well PCR plates with cut wells and distilled water. The PCR plates were placed in a culture room with light at 28°C for cultivation and later cultivated with 12 h of light and 12 h of darkness. After 7 days, the distilled water was changed to the standard nutrition of the International Rice Research Institute (IRRI) but with only 0.5-fold concentration (Yoshida et al., 1976), while keeping the PH value of Yoshida’s solution at 5.5. The nutrient solution was changed once per 3 days. After culturing for 7 days, the cultivation was changed to 0.5-fold Yoshida’s solution and later changed to one-fold Yoshida’s solution with regularly replacing the nutrient solution. When the seedlings grew to the three-leaf stage, the samples of both the control and the salt treated were extracted in 25 ml acetic acid (100 mm) at 90°C for 2 h, and 2 ml extraction was divided into two groups for sodium and potassium, respectively. 100 mm NaCl solution was added to the nutrient solution for salt stress treatment, while the control group continued to grow in the nutrient solution. Each treatment was set with three repetitions. After 10 days of treatment, samples were taken to measure the phenotypic traits. Shoot and root Na^+^ and K^+^ concentrations (RNC, SKC, and SNC) of each sample were determined by atomic absorption spectrometry (AAS, Series 2, Thermo Electron Corporation). Concentrations of sodium and potassium in shoots and roots were expressed in millimoles per gram (mMg^−1^; Qi et al., 2005; An et al., 2020). The K^+^/Na^+^ ratios in roots (RN/K) were calculated subsequently. A root scanner (Expression 1100XL) was used to analyze root traits. To reduce errors, 10 seedlings were scanned for each material, and each seedling was scanned 3 times, and the average values of TRSA (total root surface area), TRV (total root volume), and TRL (total root length) were calculated. Set up 3 biological replicates. RTRSA (relative total root surface area), RTRV (relative total root volume), RTRL (relative total root length), and RSN/K (relative ratio of Na^+^ to K^+^ concentrations in shoots) were calculated according to the following formula: relative trait value (%) = (trait value under salt stress) / (trait value under control) × 100.

### RNA Extraction and Real-Time PCR

In this study, to further determine whether candidate genes are related to ST, we first screened extreme salt-tolerant (S125) and extreme salt-sensitive (S87) rice landraces, RT-PCR of candidate genes was performed in salt-tolerant (S125) and sensitive (S87) rice landraces. Under the condition of salt stress, the time for rice seedlings to appear stress phenotype lags behind the response time of related genes. Under salt stress, the expression levels of some salt-related genes will change rapidly and then return to their original levels, resulting in undetectable changes in expression levels. Using high concentration and short time salt stress for gene expression analysis can more accurately analyze the changes of related gene expression. Therefore, in this study, the 20-day-old seedlings were treated with 200 mm NaCl for a short time (0, 3, 6 and 12 h) for gene expression analysis (Xiaoxue et al., 2019). The expression of genes at 0 h of salt stress in this study is as control data for non-stress (without salt stress). The total RNA from the shoots tissues of landraces was extracted using *steadyPure* Plsant RNA Extraction Kit (Accurate Biotechnology; An et al., 2020). The cDNA for real-time PCR was reverse-transcribed from 2 μg of total RNA using *Evo M-MLV* Reverse Transcription Reagent Master Mix (Accurate Biotechnology). According to the manufacturer’s instructions, real-time PCR was performed using SYBR Green *Pro Taq* HS kit (Accurate Biotechnology) in a real-time PCR machine (QuanStudio). Relative gene expression levels were determined using the 2^-ΔΔCt^ method (Livak and Schmittgen, 2002). Data analysis and graphing were performed using GraphPad Prism 6.02 software, and Duncan’s test was performed using SPSS. The rice actin was selected as the internal control. Primers used for qRT-PCR analysis are listed in (Supplementary Table S10).

### DNA Extraction and Candidate Gene Sequence Alignment

DNA Quick Plant System (Tiangen Biotechnology, Beijing, China) was used to extract plant genomic DNA. The target sequence was amplified with specific primers. The amplified product was purified with EasyPure PCR purification kit (Tiangen Biotech, China) and quantified with NanoDrop 8,000 spectrophotometer (Thermo Fisher Science, Waltham, MA, United States). Sequence alignment was performed with the DNAMAN software using the genes in the Nipponbare genome as a reference.

### GWAS Analysis and Haplotype Analysis

GWAS was performed by using the compressed MLM program in the Tassel5.0 software, where the model was as follows: Y = X*α* + Q*β* + K*μ* + e, where Q represents the kinship, X is genotype, Y is phenotype, while X*α* and Q*β* considered as fixed effects and K*μ* and e as random effects. The population structure K was calculated by the Admixture software, and the kinship between samples was calculated by the SPAGeDi software. The significant SNPs were identified by *p* value. To obtain high-density SNPs, we encrypted the original 67,511 SNPs and obtained 2,487,353 SNPs by the following method: the user uploads input files in oxford format (.gen/.sample) per chromosome. The imputation server utilizes chromosome recombination maps and the Rice Reference Panel (RICE-RP) haplotypes to impute the user’s data out to 5.2 M SNPs with IMPUTE2 and returns imputed results in plink binary format. The user can filter the imputed data using plink1.9 to produce a final data set of desired SNP density and composition (Wang et al., 2018b).^1^ Manhattan scatter plots and QQ plots are drawn using the “qqman” package in R software. LD heatmaps surrounding peaks in the GWAS were constructed using “LD heatmaps” in the R package (Shin et al., 2006). The haplotypes of at least three rice landraces were analyzed for phenotypic comparison. Differences in phenotypic values between alleles of each non-synonymous SNP were assessed by Student’s t tests. The sequence alignment of each gene was determined using non-synonymous SNPs associated with ST, and differences in phenotypic value between haplotypes of each gene were calculated by one-way ANOVA or Student’s *t* tests.

## Results

### Phenotypic Variation

In order to evaluate the phenotypic variation of ST in the core collection at the seedling stage, a statistical analysis was performed on 8 ST-related traits: RNC (root Na^+^ concentration), SKC (shoot K^+^ concentration), SNC (shoot Na^+^ concentration), RN/K (ratio of Na^+^ to K^+^ concentrations in roots), RTRSA (relative total root surface area), RTRV (relative total root volume), RTRL (relative total root length), and RSN/K (relative ratio of Na^+^ to K^+^ concentrations in shoots; Supplementary Table S1). The results showed that all traits showed tremendous phenotypic variation in the population. In particular, RN/K had the highest coefficient of variation, and RTRSA had the lowest coefficient of variation. Correlation analysis showed that most traits were significantly positively correlated. Significant negative correlations were observed only between SKC and RSN/K, SKC, and RN/K (Supplementary Table S2). The results showed that salt stress has different degrees of influence on the 8 ST-related traits at rice seedling stage.

### GWAS Results

**Figure 1:**
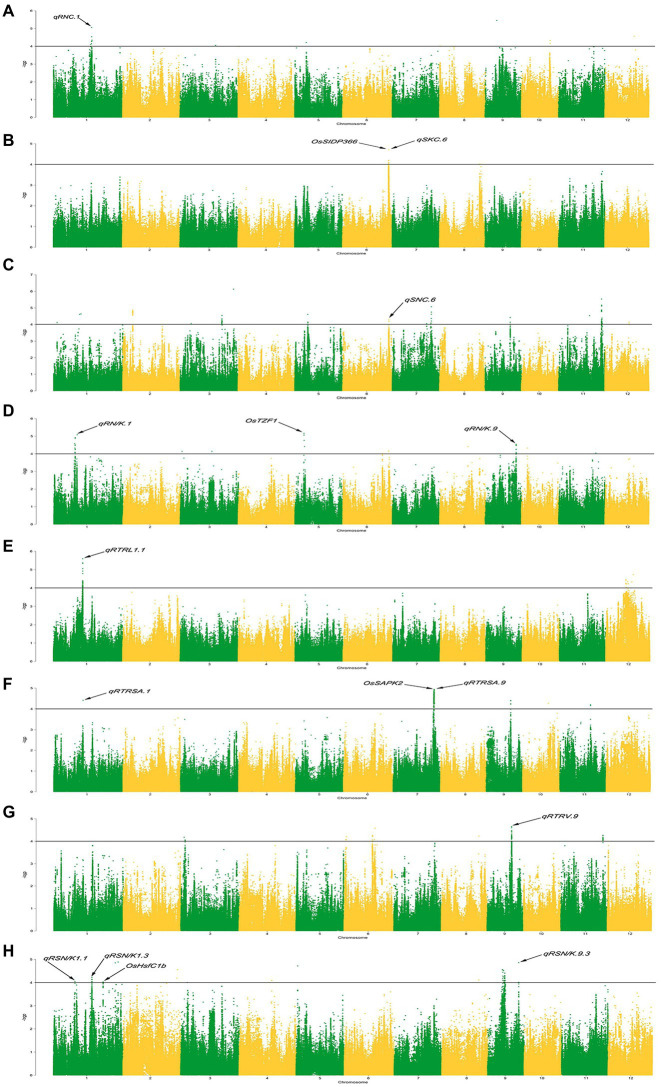
Manhattan plots of genome-wide association studies. **(A-H):** Manhattan plots for the RNC (root Na^+^ concentration), SKC (shoot K^+^ concentration), SNC (shoot Na^+^ concentration), RN/K (ratio of Na^+^ to K^+^ concentrations in roots), RTRSA (relative total root surface area), RTRV (relative total root volume), RTRL (relative total root length), and RSN/K (relative ratio of Na^+^ to K^+^ concentrations in shoots).

In previous studies, the rice core collection was sequenced using the Specific-Locus Amplified Fragment Sequencing(SLAF-seq)approach and 67,511 SNPs were obtained (Zhao et al., 2018). Based on them, 2,487,353 SNPs were further obtained by the imputation method of (Wang et al., 2018b). With consideration of the population structure and kinship, the MLM (+Q + K) model and 2,487,353 SNPs were used to perform GWAS on eight traits related to ST with the high density SNPs set, and the results are presented in the form of Manhattan plots (Figure 1) and QQ plots (Supplementary Figure S1). With a significant threshold of *p* < 0.0001, 843 significant SNPs were detected (Supplementary Table S3), which were unevenly distributed on the 12 chromosomes. The most significant position with the largest contribution rate is located on chromosome 1, which can explain 28.11% of the phenotypic variation.

### QTLs for ST at the Seedling Stage

**Figure 2:**
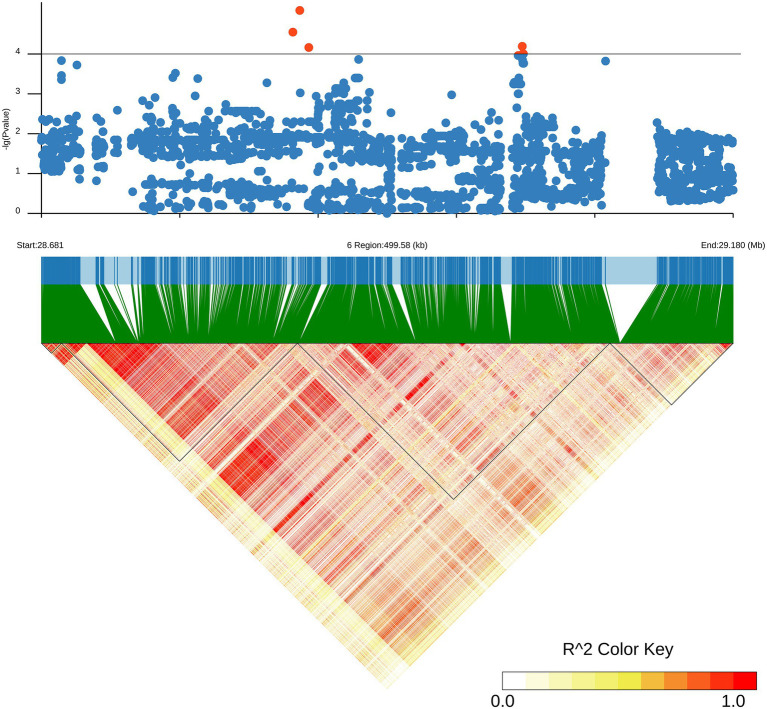
Local Manhattan plot (upper) and LD heatmap (lower) surrounding the lead SNP for SKC on chromosome 6.

ST-related QTLs are defined by the decay distance of linkage disequilibrium (LD). Previous studies had shown that the decay distance of LD is 500 kb (Zhao et al., 2018). Therefore, a region was considered as one QTL if it had more than one SNP with *p* < 0.0001 within the LD decay distance. We named salt tolerance QTLs with reference to the method proposed by Mccouch et al. (1997). In total, 65 QTLs were identified significantly associated with ST (Figure 2). There are 1–13 of QTLs on each chromosome, and each QTL contains 1–379 SNPs. These QTLs explained 13.47 to 28.11% of the phenotype variation (Supplementary Table S4).

For RNC, we detected 8 QTLs located on chromosomes 1, 3, 5, 8, 9, 10, and 12 under salt stress, which could explain 14.83–23.96% of the phenotypic variation. Under salt stress, we detected 14 QTLs for SNC on chromosomes 1, 2, 3, 5, 6, 7, 9, 11, and 12, which can explain 13.91–23.61% of the phenotypic variation. For RN/K under salt stress, ten QTLs were detected on chromosomes 1, 3, 5, 6, 8, 9, 10, and 11, which could explain 13.75–27.74% of the phenotypic variation. For RTRV under salt stress, 8 QTLs were detected on chromosomes 3, 6, 8, 9, and 11, which could explain 13.47–20.52% of the phenotypic variation. Under salt stress, we detected 13 QTLs for SN/K on chromosomes 1, 2, 3, 4, 5, 8, and 9, which could explain 13.67–21.14% of the phenotypic variation. For SKC under salt stress, two QTLs were detected on chromosomes 6 and 8, which could explain 14.38–17.94% of the phenotypic variation. Under salt stress, we detected 5 QTLs for SKC on chromosomes 1 and 12, which can explain 14.46–28.11% of the phenotypic variation. For TRSA under salt stress, 5 QTLs were detected on chromosomes 1, 7, 9, 10, and 11, which can explain 14.33–23.10% of the phenotypic variation.

### Co-localization of QTLs Under Salinity Stress Conditions

Through association mapping analysis, there are 6 genomic regions containing co-localization QTLs. We defined co-localization QTLs as *qTL1*, *qTL2*, *qTL3*, *qTL4*, *qTL5*, and *qTL6*, respectively (Table 1). The most complicated is the co-localization region of chromosome 6, i.e., *qTL4*, which is composed of 3 QTLs and has a large overlap region. The remaining 5 co-localization regions are all composed of 2 QTLs.

**Table 1:** Co-localization of QTLs at seeding stage.

QTL	Chr	Position/interval (Mbp)	No. of Co-located QTLs	No. of Associated SNPs	Co-located QTLs
*qTL1*	1	12.98–13.34	2	18	*qRN/K.1,qRSN/K1.1*
*qTL2*	1	18.01–18.26	2	73	*qRTRL1.1,qRTRSA.1*
*qTL3*	1	23.28–24.03	2	17	*qRNC.1,qRSN/K1.3*
*qTL4*	6	28.76–29.01	3	14	*qSKC.6,qRN/K.6,qSNC.6*
*qTL5*	9	15.43–15.53	2	67	*qRTRV.9,qRTRSA.9*
*qTL6*	9	19.50–19.54	2	11	*qRN/K.9,qRSN/K.9.3*

### Identification of Candidate Genes for ST

**Figure 3:**
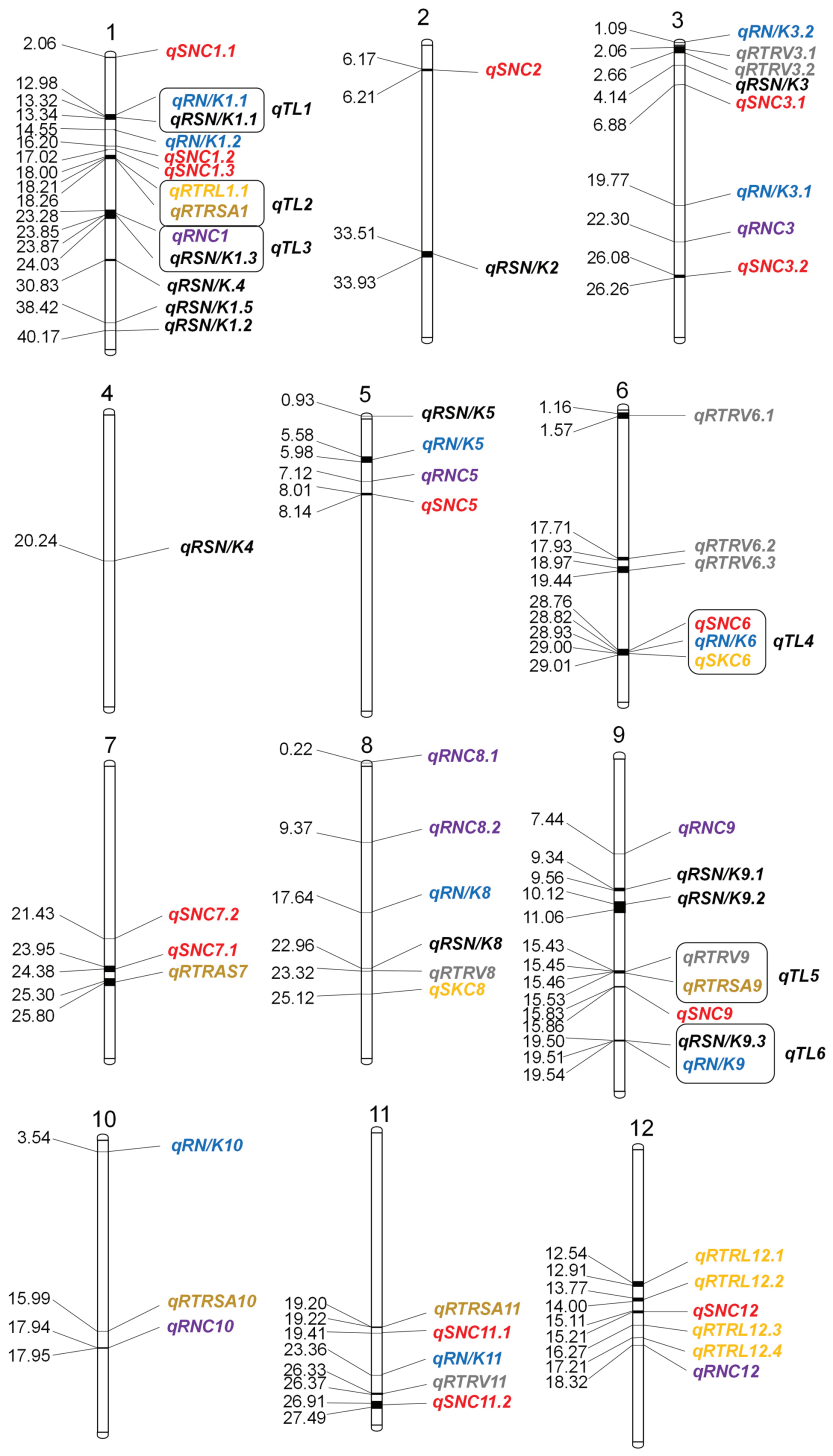
Distribution of GWAS-based detected QTLs on 12 chromosomes in rice. Distances on the map are in Mbp.

Since *qTL4* is composed of 3 QTLs with 14 significant SNPs, it was chosen for further analysis. The *qTL4* contains the highest peak SNP (Chr6_28930159) at approximately 28.93Mbp, which can explain 14.38–17.94% of the phenotypic variation. According to the LD decay analysis of the population, the 250 kb upstream and downstream around the peak SNP were designated as the searching range of candidate genes as shown by the LD heat map (Figure 3). Through the Rice Annotation Project database,^2^ ninety-four genes were found in this region (Supplementary Table S5). It includes 50 functional annotation genes, 24 expressed proteins, 17 transposon proteins, and 3 hypothetical proteins. Among them, a known salt tolerant gene (*OsSIDP366*) was found in this region, which was located in the interval between *qSKC.6* and *qSNC*.6. Besides it, *qRN/K1.1* and *qRSN/K1.1* also were co-localization, which were detected in the interval of *qTL6* and related to ratio of Na^+^ to K^+^ concentrations. The *qTL6* contains the highest peak SNP (Chr9_19518843) at approximately 19.51Mbp, which can explain 13.75–16.92% of the phenotypic variation. In addition, we also found a SNP Chr6_28822519 in the *qRN/K.6* interval with a significant threshold of *p* = 0.000069293, which could explain 17.49% of the phenotypic variation, indicating that there may be new salt resistance-related genes in this interval. Based on gene functional annotation and GO enrichment analysis^3^ (Supplementary Table S6), we screened and found 11 out of 50 functional genes which are related to stress response or metabolism process-related genes were salt tolerance-related candidate genes (Supplementary Table S7).

### Haplotype Analysis of Candidate Genes

**Figure 4:**
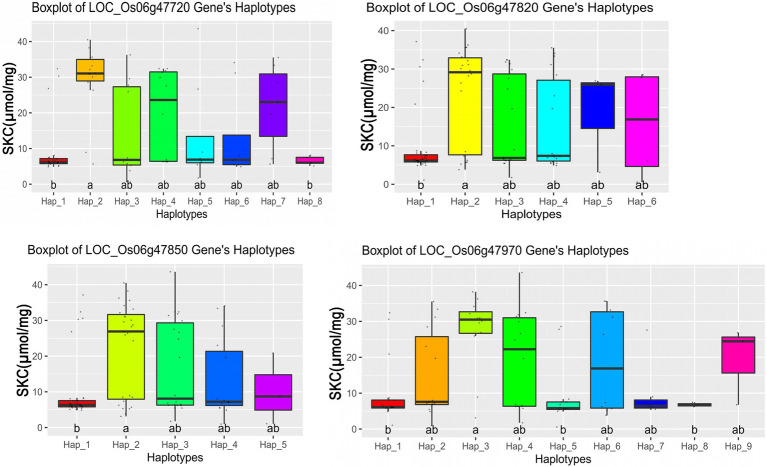
Boxplots for the entire population based on the haplotypes (Hap) for four candidate genes, where the a and b are ranked by Duncan’s test at *p* < 0.05.

We further performed haplotype analysis on the non-synonymous mutant SNPs and the promoter SNPs in the exon region of the 11 candidate genes and detected significant differences among different major haplotypes based on the haplotypes with all non-synonymous SNPs. It was found that the haplotypes of the 4 candidate genes had significant differences (Table 2), and each candidate gene had a different number of non-synonymous SNPs (Supplementary Table S8). The haplotype analysis showed that the four candidate genes were significantly associated with SKC (shoot K^+^ concentration), and the SKC of different haplotypes were also significantly different (Figure 4). These findings suggest that the four candidate genes (*LOC_Os06g47720*, *LOC_Os06g47820*, *LOC_Os06g47850, and LOC_Os06g47970*) may be involved in the regulation of salt tolerance at seedling stage in rice.

**Table 2:** Candidate genes.

Related traits	Gene ID	Position (Mbp)	Gene description
SKC	LOC_Os06g47720	28,874,838–28,878,068	Serine/threonine-protein kinase BRI1-like 2 precursor, putative, expressed
	LOC_Os06g47820	28,941,271–28,943,703	Protein kinase domain containing protein, expressed
	LOC_Os06g47850	28,958,626–28,960,418	Zinc finger family protein, putative, expressed
	LOC_Os06g47970	29,002,279–29,003,541	Protein of unknown function DUF1517 domain containing protein

### Identification of Candidate Genes by Gene Expression

**Figure 5:**
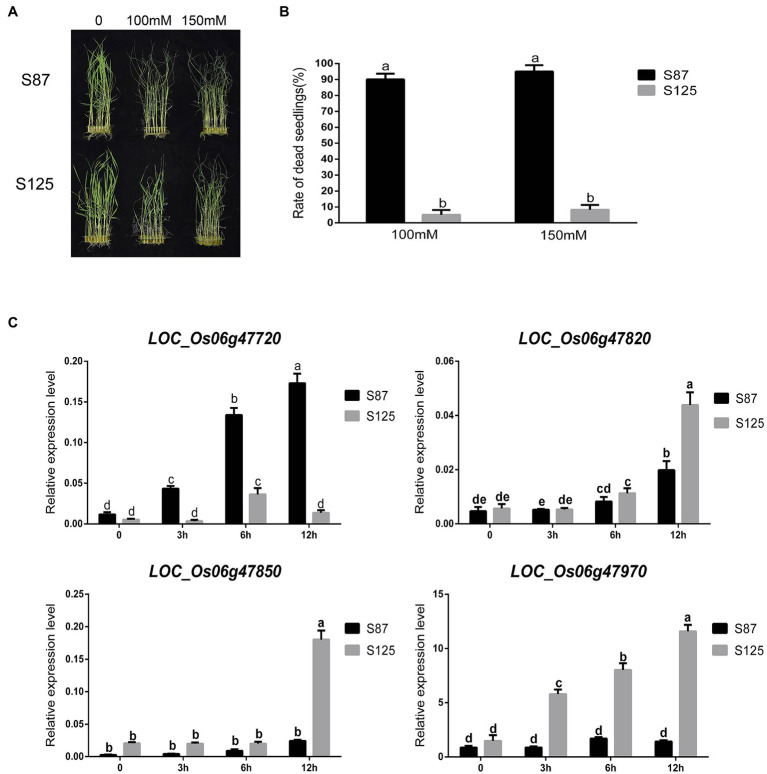
**(A)** Phenotypes of S87 (salt sensitive variety) and S125 (salt tolerant variety) at different salt concentrations. **(B)** Statistical analysis of S87 and S125 for dead seedling rate, where the a and b are ranked by Duncan’s test at *p* < 0.05. **(C)** changes in relative gene expression level of four candidate genes at different times (0, 3 h, 6 h, 12 h) after salinity stress in different rice cultivars, where the a, b, c, d, and e are ranked by Duncan’s test at p < 0.05.

To further determine whether the aforementioned 4 candidate genes are related to ST, gene expression assays for the 4 candidate genes were conducted in salt tolerant (S125) and sensitive (S87) rice landraces. We performed a statistical analysis on the dead seedling rates in S87 and S125 under salt stress to further verify their differences in salt tolerance (Figure 5). There were significant differences in the dead seedling rates of S87 and S125 under the two salt stress concentrations, indicating that they had significant differences in salt tolerance. Therefore, S87 and S125 were selected for subsequent analysis of candidate genes expression. The gene expression level of *LOC_Os06g47720* in S87 showed a continuous increase trend from 0–12 h and was higher than S125 for all tested time points. The gene expression level of *LOC_Os06g47720* in S87 was nearly 10-fold higher than that in S125 after 12 h of salt stress treatment. The opposite expression pattern was observed for *LOC_Os06g47820*, *LOC_Os06g47850*, and *LOC_Os06g47970*, where the gene expression levels in S125 were higher than those in S87 for all tested time points after salt stress treatment. Among them, the gene expression levels of *LOC_Os06g47850* and *LOC_Os06g47970* in S125 were nearly 10-fold higher than those in S87 after 12 h of salt stress treatment.

### Sequence Analysis of the Candidate Genes

To determine the association between candidate genes and ST phenotypes, four candidate genes were sequenced for the two rice landraces S125 and S87 (Supplementary Figure S1) and the sequence alignment was generated using DNAMAN. *LOC_Os06g47720* has 29 mutation sites in the two ST extreme genotypes, which lead to 11 amino acids changed in the S125, while translation was terminated early in the S125. For *LOC_Os06g47820*, it showed that the two genotypes had 17 mutation sites and 14 amino acids changed. *LOC_Os06g47970* has a pair of base substitutions in S125, which caused the glycine to change into the asparagine. *LOC_Os06g47850* has no sequence difference between the two genotypes.

## Discussion

### Phenotypic Variation and Prospects of the Core Collection of Rice Landraces

Artificial directional domestication and selection have led to the reduction of genetic diversity in many crops such as rice, and many excellent resistance gene resources have been gradually lost in common cultivated rice. Some landraces are less affected by artificial selection and contain more genetic diversity and resistance genes. The core collection of rice landraces was constructed from a total of 7,128 accessions of rice landraces from the Ting’s rice collection (Li et al., 2011) and came from all over China as well as some main rice cultivation countries. It provides an important gene pool of genetic diversity and beneficial genes for rice breeding. For example, several QTLs for aluminum tolerance and cold tolerance were identified in the core collection (Song et al., 2018; Zhao et al., 2018). In this study，8 traits related to ST showed tremendous phenotypic variation in the core collection, which suggested that salt stress has different degrees of influence on the ST-related traits at the seedling stage, and these phenotypes could be used for GWAS. Furthermore, a total of 65 QTLs significantly associated with salt tolerance were identified by GWAS. These results further verified that the core collection of rice landraces contains abundant resistance gene resources to biotic stress and abiotic stress and is a precious germplasm resource with great prospect for mining and utilization of beneficial genes for rice breeding.

### Comparison of QTLs With Previous Studies

GWAS is a promising method for QTL fine-mapping in plants in response to abiotic stress. At present, many QTLs related to salt stress have been identified. We compared the previously reported QTLs and found that a total of 12 previously reported QTLs for ST in this study were overlapped or close to the range of previously mapped QTLs for ST.

For example, under stress conditions 151 trait–marker associations were identified that were scattered in 29 genomic regions on 10 chromosomes of rice (Nayyeripasand et al., 2021). Some of the QTLs are consistent with or close to our mapped QTL regions. The QTL *qSNC1.1* on chromosome 1 coincides with the reported QTL *qRDWn1.1* (root dry weight); the QTL *qRNC1* is located in the region of the reported QTL *qRDWs1.1* (root dry weight). The QTLs *qRTRL12.1* and *qRTRL12.2* on chromosome 12 are located in the region of the reported QTL *qSLn12.1* (shoot length). The QTL *qRTRL12.4* is 320kbp away from the previously reported QTL *qRDWs12.1* (root dry weight), and the QTL *qRNC12.1* (root fresh weight) is 140 kb apart from the previously reported QTL *qRFWs12*. The QTL *qRN/K8* on chromosome 8 is located in the previously reported QTL *qRFWn8.1* (root fresh weight) interval. Chen et al. (2020) identified 23 QTLs with different salt tolerance indexes through GWAS. Some of the QTLs coincide with our regions. The QTL *qRSN/K1.2* is located in the region of previously reported QTL *qSIS1* (salt injury score) and *qSNaC1.2* (shoot Na^+^ concentration), while the QTL *qSNC2* on chromosome 2 is within the interval of previously reported *qRRDW2* (relative root dry weight). The QTL *qSKC8* is located in the interval of previously reported *qRSKC8* (relative shoot K^+^ concentration) and *qRNaC8* (root Na^+^ concentration). Zhang et al. (2020) used the 55 K rice SNP array to genotype the entire population and four parents, and a total of 7 salt-tolerant QTLs were detected. Some of the QTLs coincide with or close to our regions. The QTL *qRSN/K1.5* is about 174kbp away from the previously reported QTL *qSLST1* (shoot length under salt stress treatment) and is located in the intervals of previously reported QTL *qRDSW1* (relative dry shoot weight) and *qRB1* (relative bio-mass). The QTL *qSNC9* on chromosome 9 is 430 kb away from the previously reported QTL *qRL-R9.1* (root length), and the QTL *qRNC9* is 430kbp away from the previously reported QTL *qDSW9* (dry shoot weight) and *qBST9* (biomass under salt treatment). It can be seen that some of the QTLs we mapped by GWAS were overlapped with or were close to the previously reported QTLs, which demonstrates that our GWAS mapping results are rather accurate.

In addition, 52 newly identified QTLs for ST were also found in this study. Among them, *qRN/K1.1* and *qRSN/K1.1* were located on chromosome 1 between 12.98–13.34Mbp and can explain 13.75–16.92% of phenotypic variation. The QTL *qRTRV.9* and *qRTRSA.9* between 15.43–15.53Mbp on chromosome 9 can explain 13.47–17.14% of the phenotypic variation. The QTL *qRN/K.9* and *qRSN/K9.3* between 19.50 and 19.54 Mb on chromosome 9 can explain 16.65–21.14% of phenotypic variation. These QTLs are newly discovered and need to be further examined in the future.

Moreover, we also compared the cold-tolerant and aluminum-tolerant QTLs previously mapped using the same core collection. Some QTLs are overlapped or are similar to the QTL regions for ST in this study. Compared with the previously located QTLs for cold tolerance, the QTL *qRSN/K4* in this study on chromosome 4 is about 200kbp away from the previously located cold-tolerant SNP (Chr4_20440388). The QTL *qRTRV6.1* on chromosome 6 contains the previously located cold-tolerant SNP (Chr6_1320300). The QTL *qRNC8.1* on chromosome 8 is about 151kbp away from the previously located cold-tolerant SNP (Chr8_374729). The QTL *qRN/K10* on chromosome 10 is about 18kbp away from the previously located cold-tolerant SNP (Chr10_3365663; Song et al., 2018). Compared with the previously located QTLs for aluminum tolerance, the QTL *qRN/K1.1* and *qRSN/K1.1* on chromosome 1 are about 330kbp away from the previously located QTL *qALT1.3*. The QTL *qRNC1* and *qRSN/K1.3* on chromosome 1 are about 400kbp away from the previously located QTL *qALT1.5*. The QTL *qRTRSA7* on chromosome 7 is about 84kbp away from the previously located QTL *qALT7.2*. The QTL region for *qRSN/K9.2* contains the previously located QTL *qALT9.1* (Zhao et al., 2018). These results indicate that the candidate genes in these intervals may have pleiotropic effects. It also indicates that the core collection contains a wealth of excellent resistance genes to biotic and abiotic stress.

### Comparison of the QTL Locations With the ST Genes

We also compared the QTL locations with the genes known to be related to ST.^4^ Twenty-three ST genes were found to co-localize with our QTLs (Table 3). Three ST genes were found close to the mapped QTLs related to RNC in this study. Seven ST genes were found close to the mapped QTLs related to SNC. One ST gene was found in the QTL interval related to RN/K. Three ST genes were found close to the QTLs related to RTRV. Four ST genes were found close to the mapped QTLs related to RSN/K. Three ST genes were found close to the mapped QTLs related to SKC. One ST gene was found close to the QTLs related to RTRL. One ST gene was found close to the QTLs related to RTRAS. In short, these ST genes are located in or close to the relevant QTLs interval (with a searching range of +/− 250 kb). Among them, the QTL *qTL4* region is composed of *qSKC.6*, *qRN/K.6*, and *qSNC.6* and the overlapping regions are dense. A known ST gene *OsSIDP366* was found in the region of *qTL4*, where a candidate gene and *OsSIDP366* both contain the DUF domain. These findings support the reliability of the mapping results in this study.

**Table 3:** Known ST genes surrounding the mapped QTLs.

Related traits	QTLs	Chr	Position (Mbp)	Known genes
RNC	*qRNC5*	5	7.13	*OsCYP51G3* Xia et al. 2015 *OsPYL* Tian et al. 2015
	*qRNC9*	9	7.44	*OsMYBc* Wang et al. 2015
SNC	*qSNC1.1*	1	2.07	*OsRAV2* Duan et al. 2016 *HSP17.0* Ham et al. 2013
	*qSNC2*	2	6.18–6.21	*OsGIRL1* Park et al. 2014
	*qSNC3.1*	3	6.88	*DSM3* Du et al. 2011
	*qSNC9*	9	15.83–15.86	*OsDSG1* Park et al. 2010 *OsBIERF1* Cao et al. 2006
	*qSNC11.2*	11	26.92–27.49	*OsJAMyb* Yang et al. 2020
RN/K	*qRN/K5*	5	5.59–5.98	*OsTZF1* Jan et al. 2013
RTRV	*qRTRV3.1*	3	2.06	*ONAC022* Hong et al. 2016
	*qRTRV3.2*	3	2.66–2.67	*OsAP23* Zhuang et al. 2013
	*qRTRV6.1*	6	1.16–1.58	*OsSIK1* Ouyang et al. 2010
RSN/K	*qRSN/K1.4*	1	30.84	*OsHsfC1b* Schmidt et al. 2012
	*qRSN/K1.5*	1	38.43	*OsNAC6* Chung et al. 2009
	*qRSN/K2*	2	33.52–33.93	*OsEREBP1* Jisha et al. 2015
	*qRSN/K3*	3	4.15	*OsJAZ9* Wu et al. 2015
SKC	*qSKC6*	6	28.76–29.01	*OsSIDP366* Guo et al. 2016
	*qSKC8*	8	25.12	*OsDOG* Giri et al. 2011 *OsXylT* Takano et al. 2015
RTRL	*qRTRL1.1*	1	18.01–18.26	*OsMEK1* Wen et al. 2002
RTRAS	*qRTRAS7*	7	25.30–25.80	*OsSAPK2* Xu et al. 2013

### Promising Candidate Genes for ST in Rice

The salinity tolerance in rice seedling is majorly governed by root and shoots Na^+^/K^+^ ratio. The lower Na^+^/K^+^ ratio provides protection against the toxic effects of Na^+^, hence, tolerant to salt stress. Therefore, maintaining intracellular Na^+^/K^+^ homeostasis is a key factor in determining the survival ability of plants in response to salt stress (Yang and Yan, 2018). In this study, we found that *qSKC6*, *qSNC6*, and *qRN/K.6* were co-localization and 4 candidate genes (*LOC_Os06g47720*, *LOC_Os06g47820*, *LOC_Os06g47850*, and *LOC_Os06g47970*) were detected in the interval of *qTL4* which are all related to ratio of Na^+^ to K^+^ concentrations; therefore, we chose the four candidate genes for further study. Besides, *qRN/K1.1* and *qRSN/K1.1* also were co-localization and detected in the interval of *qTL6* which are related to Na^+^ and K^+^, the *qTL6* contains the highest peak SNP (Chr9_19518843). Therefore, searching for candidate genes in the interval of *qTL6* is also the focus of our next study.

To do it, we first checked the expression profiles of these four candidate genes from the Encyclopedia of Rice Transcriptome (TENOR) database.^5^ According to the TENOR database (Supplementary Figure S2), the candidate gene *LOC_Os06g47720* has a higher expression level under salt stress conditions. The gene annotation of *LOC_Os06g47720* is a serine threonine protein kinase BRI1-like 2 precursor. BRI1 (protein brassinosteroid insensitive 1) is the receptor kinase of BR (brassinosteroids), located on the cell membrane, and is a leucine-rich repeat (LRR) receptor-like serine/threonine kinase on the cell surface, which is crucial in the BR signaling pathway. BR is a plant steroid hormone, which plays a key role in growth and response to abiotic and biotic stress (Zhao et al., 2019; Ma et al., 2021). The plants adapt to various environmental stresses by changing their physiological and molecular processes, which are coordinated with changes of the levels on hormones (including brassinosteroids) in plant externally and internally (Bilal et al., 2021). The response of BR under salt stress may be mediated by BRI1, inhibiting the degradation of the endoplasmic reticulum to combat salt stress (Cui et al., 2012). BR signaling also counters salt stress by signaling cascades or initiating ethylene biosynthesis (Planas-Riverola et al., 2019). In this study, the expression level of *LOC_Os06g47720* in S87 is much higher than that in S125, and *LOC_Os06g47720* had far more mutation sites in S87 than in S125. By comparison of amino acid sequences, it also revealed that only the amino acids in S87 were changed and the translation was terminated in S125. These findings suggest that *LOC_Os06g47720* may be involved in the regulation of ST through the BR pathway.

The candidate gene *LOC_Os06g47820* is receptor-like kinases, which has the highest expression level under ABA treatment conditions (Supplementary Figure S2). ABA can coordinate with hormones such as auxin, gibberellin (GA) and cytokinin (CK) to regulate the response of plants to salt stress (Yu et al., 2020). Receptor-like kinases (RLKs) are a large family of proteins that exist on the surface of plant cell membranes. Their basic function is to transmit regulatory signals on the cell surface. Plant receptor-like protein kinases occupy important metabolic positions, and rice has about 1,130 RLK genes (Quynh-Nga et al., 2015). Plant RLKs are composed of intracellular, extracellular, and transmembrane regions (Ye et al., 2017). Receptor-like protein kinase RLK is widely involved in cell signal transduction and plant response to stress (Lemmon and Schlessinger, 2010). In recent years, several researchers studied the important role of RLKs in optimizing the response of plants to salt stress and other abiotic stresses (Zhou et al., 2018; Lin et al., 2020). In this study, the expression level of *LOC_Os06g47820* in S125 was found much higher than that in S87. The gene had much more mutation sites in S125 than in S87. There are more changes in amino acids in S125 than in S87. These findings indicate that *LOC_Os06g47820* is a candidate gene that may be involved in the regulation of ST in rice.

The candidate gene *LOC_Os06g47850* encodes a zinc finger family protein. The zinc finger protein (ZFP) family is widely distributed in eukaryotic genomes and is one of the most important transcription factors, which plays an important role in plant growth and development and abiotic stress response (Mukhopadhyay et al., 2004; Sakamoto, 2004). More than 60 transcription factor families have been reported in plants (Iuchi, 2001). *LOC_Os06g47850* has the highest expression level under cold stress conditions (Supplementary Figure S2). In our study, the expression level of *LOC_Os06g47820* in S125 was much higher than that in S87. DNA sequence alignment results showed that the gene has no mutation sites. Therefore, the mechanism of this candidate gene remains to be elucidated.

The candidate gene *LOC_Os06g47970* encodes a DUF1517 (domains of unknown function, DUF) which are a class of proteins whose functions have not been characterized and account for about 25% of the total protein family (Mudgal et al., 2015). According to the TENOR database (Supplementary Figure S2), *LOC_Os06g47970* has the highest expression level under drought stress and as we know that salinity usually occurs at the same time as drought stress (Hu et al., 2006). In recent years, an increasing number of studies were conducted on the regulation of different DUFs family genes involved in plant growth and development and plant response to stress (biotic and abiotic stress; Li et al., 2018; Lv et al., 2019). We found a DUFs gene in the *qTL4*, i.e., *OsSIDP366*, which is a stress-induced DUF1644 protein and contains a DUF1644 domain, a C2H2 and a ring finger domain. *OsSIDP366* is expressed in multiple tissues. The expression is higher in young roots, mature leaves, and leaf sheaths, but lower or no expression in internodes, mature seeds, lemmas and glumes. High salt and drought treatments can induce the expression of *OsSIDP366*. *OsSIDP366* may positively regulate salt and drought resistance in rice (Guo et al., 2016). In addition, we also found that the homologous gene *AT5G57345* in *Arabidopsis*, a single copy gene, is localized to ER and expressed in the whole plant and induced expression in response to abiotic stress. Although the function of *AT5G57345* is unclear, overexpression can lead to increased tolerance to abiotic stress and increased ascorbic acid content (Bu et al., 2016). In addition, we also found that the expression level of *LOC_Os06g47970* in S125 was much higher than that in S87. Sequence analysis found that *LOC_Os06g47970* only had a pair of base substitutions in S125, i.e., one amino acid was changed in S125, while no change in S87. Therefore, *LOC_Os06g47970* might be involved in the regulation of ST in rice.

This study lays a foundation for the functional analysis of the candidate genes in the regulation of salt tolerance in rice and the enrichment of the rice salt tolerance regulatory network. In addition, our newly discovered QTLs also lay the foundation for further research on the ST mechanism in rice in the future. The salt tolerance-related candidate genes and QTLs would provide important resources for molecular breeding and functional analysis of the salt tolerance during the seedling stage of rice. However, the four candidate genes identified in this study which involved in the regulation of salt stress in rice need further research and verification. In addition, the mechanism and the regulation pathway for these genes under salt stress in rice still need to be clarified. In future, the function of candidate genes related to salt tolerance will be studied by Crispr-Cas9 technology, which will help to precisely uncover the mechanisms of salinity tolerance at molecular level.

## Conclusion

Eight seedling-stage salt-related traits within a core collection of rice landraces were evaluated under salinity stress (100 mm NaCl) in a growth chamber, and abundant phenotypic variations were observed for these traits. With 2,487,353 SNPs derived from an enrichment of 67,511 SNPs from SLAF-seq, GWAS was performed for the eight traits related to ST with a mixed linear model. In total, 65 QTLs were identified significantly associated with eight ST traits. These QTLs explained 13.47 to 28.11% of the phenotype variation. There are 8 QTLs for RNC, 14 QTLs for SNC, 10 QTLs for RN/K, 8 QTLs for RTRV, 13 QTLs for SN/K, 2 QTLs for SKC, 5 QTLs for SKC, and 5 QTLs for TRSA. Several QTLs in this study were overlapped with or were close to the previously reported candidate genes or QTLs related to ST. There are 6 genomic regions containing co-localization QTLs (qTL1 – qTL6). Among them, a co-localization QTL qTL4 associated with the SKC, RN/K and SNC on chromosome 6, which explained 14.38–17.94% of phenotypic variation, was selected for further analysis. According to haplotype analysis, qRT-PCR analysis, and sequence alignment, it was finally determined that 4 candidate genes (*LOC_Os06g47720*, *LOC_Os06g47820*, *LOC_Os06g47850*, and *LOC_Os06g47970*) were related to ST. The results provide useful candidate genes for marker-assisted selection for ST in the rice molecular breeding programs.

## Data Availability Statement

The datasets presented in this study can be found in online repositories. The names of the repository/repositories and accession number(s) can be found in the article/Supplementary Material.

## Author Contributions

MZ and WC designed the study. MZ, XW, SG, CS, YL, and DM performed data analyses. XW and JS performed GWAS and statistical analyses. MZ, XW, and CS performed searching candidate genes/QTLs. XW wrote the paper. JL provided the rice core collection. MZ and JL revised the manuscript. All authors read and approved the final manuscript.

## Funding

This work was supported by LiaoNing Revitalization Talents Program (XLYC2008025).

## Conflict of Interest

JL is employed by Strube Research GmbH & Co.

The remaining authors declare that the research was conducted in the absence of any commercial or financial relationships that could be construed as a potential conflict of interest.

## Publisher’s Note

All claims expressed in this article are solely those of the authors and do not necessarily represent those of their affiliated organizations, or those of the publisher, the editors and the reviewers. Any product that may be evaluated in this article, or claim that may be made by its manufacturer, is not guaranteed or endorsed by the publisher.

## Supplementary Material

The Supplementary Material for this article can be found online at: https://www.frontiersin.org/articles/10.3389/fpls.2022.847863/full#supplementary-material

Supplementary Figure S1 QQ plots of genome-wide association studies for the eight traits related to ST. A-H: QQ plots for RNC, SKC, SNC, RN/K, RTRL, RTRSA, RTRV, and RSN/K.

Supplementary Figure S2 **(A–C)** DNA sequence analysis and amino acid sequence analysis for three candidate genes.

Supplementary Figure S3 Expression profiles in rice seedling under the various environmental conditions.

## Footnotes

^1^ http://rice-impute.biotech.cornell.edu

^2^ http://rice.uga.edu

^3^ http://www.bioinformatics.com.cn

^4^ http://qtaro.abr.affrc.go.jp

^5^ http://tenor.dna.affrc.go.jp/

## References

[ref1] An H. Liu K. Wang B. Tian Y. Ge Y. Zhang Y.. (2020). Genome-wide association study identifies QTLs conferring salt tolerance in rice. Plant Breed. 139, 73–82. doi: 10.1111/pbr.12750

[ref2] Bilal H. M. Noreen Z. Kiran Z. Ali R. Aaliya K. Kanval S.. (2021). Brassinosteroids: molecular and physiological responses in plant growth and abiotic stresses. Plant Stress 2:100029. doi: 10.1016/j.stress.2021.100029

[ref3] Bu Y. Sun B. Zhou A. Zhang X. Takano T. Liu S. (2016). Overexpression of AtOxR gene improves abiotic stresses tolerance and vitamin C content in *Arabidopsis thaliana*. BMC Biotechnol. 16:69. doi: 10.1186/s12896-016-0299-0, PMID: 27717369 PMC5055693

[ref4] Cao Y. Song F. Goodman R. M. Zheng Z. (2006). Molecular characterization of four rice genes encoding ethylene-responsive transcriptional factors and their expressions in response to biotic and abiotic stress. J. Plant Physiol. 163, 1167–1178. doi: 10.1016/j.jplph.2005.11.004, PMID: 16436304

[ref5] Chen T. Zhu Y. Chen K. Shen C. Zhao X. Shabala S.. (2020). Identification of new QTL for salt tolerance from rice variety Pokkali. J. Agron. Crop Sci. 206, 202–213. doi: 10.1111/jac.12387

[ref6] Chung P. J. Kim Y. S. Jeong J. S. Park S.-H. Nahm B. H. Kim J.-K. (2009). The histone deacetylase OsHDAC1 epigenetically regulates the OsNAC6 gene that controls seedling root growth in rice. Plant J. 59, 764–776. doi: 10.1111/j.1365-313X.2009.03908.x, PMID: 19453457

[ref7] Cui F. Liu L. Li Q. Yang C. Xie Q. (2012). UBC32 mediated oxidative tolerance in Arabidopsis. J. Genet. Genomics 39, 415–417. doi: 10.1016/j.jgg.2012.05.005, PMID: 22884097

[ref8] Du H. Liu L. You L. Yang M. He Y. Li X.. (2011). Characterization of an inositol 1,3,4-trisphosphate 5/6-kinase gene that is essential for drought and salt stress responses in rice. Plant Mol. Biol. 77, 547–563. doi: 10.1007/s11103-011-9830-9, PMID: 22038091

[ref9] Duan Y. B. Li J. Qin R. Y. Xu R. F. Li H. Yang Y. C.. (2016). Identification of a regulatory element responsible for salt induction of rice OsRAV2 through ex situ and in situ promoter analysis. Plant Mol. Biol. 90, 49–62. doi: 10.1007/s11103-015-0393-z, PMID: 26482477

[ref10] Giri J. Vij S. Dansana P. K. Tyagi A. K. (2011). Rice A20/AN1 zinc-finger containing stress-associated proteins (SAP1/11) and a receptor-like cytoplasmic kinase (OsRLCK253) interact via A20 zinc-finger and confer abiotic stress tolerance in transgenic Arabidopsis plants. New Phytol. 191, 721–732. doi: 10.1111/j.1469-8137.2011.03740.x 21534973

[ref11] Guo C. Luo C. Guo L. Li M. Guo X. Zhang Y.. (2016). OsSIDP366, a DUF1644 gene, positively regulates responses to drought and salt stresses in rice. J. Integr. Plant Biol. 58, 492–502. doi: 10.1111/jipb.12376, PMID: 26172270

[ref12] Ham D.-J. Moon J.-C. Hwang S.-G. Jang C. S. (2013). Molecular characterization of two small heat shock protein genes in rice: their expression patterns, localizations, networks, and heterogeneous over expressions. Mol. Biol. Rep. 40, 6709–6720. doi: 10.1007/s11033-013-2786-x, PMID: 24078098

[ref13] Hirschhorn J. N. Daly M. J. (2005). Genome-wide association studies for common diseases and complex traits. Nat. Rev. Genet. 6, 95–108. doi: 10.1038/nrg1521 15716906

[ref14] Hong Y. Zhang H. Huang L. Li D. Song F. (2016). Overexpression of a stress-responsive NAC transcription factor gene ONACO22 improves drought and salt tolerance in Rice. Front. Plant Sci. 7:4. doi: 10.3389/fpls.2016.00004 26834774 PMC4722120

[ref15] Hu Y. Burucs Z. Schmidhalter U. (2006). Short-term effect of drought and salinity on growth and mineral elements in wheat seedlings. J. Plant Nutr. 29, 2227–2243. doi: 10.1080/01904160600975111

[ref400] Iuchi S. (2001). Three classes of C2H2 zinc finger proteins. Cell. Mol. Life Sci. 58, 625–635. doi: 10.1007/PL00000885, PMID: 11361095 PMC11146492

[ref17] Jahan N. Zhang Y. Lv Y. Song M. Zhao C. Hu H.. (2020). QTL analysis for rice salinity tolerance and fine mapping of a candidate locus qSL7 for shoot length under salt stress. Plant Growth Regul. 90, 307–319. doi: 10.1007/s10725-019-00566-3

[ref18] Jan A. Maruyama K. Todaka D. Kidokoro S. Abo M. Yoshimura E.. (2013). OsTZF1, a CCCH-tandem zinc finger protein, confers delayed senescence and stress tolerance in Rice by regulating stress-related genes. Plant Physiol. 161, 1202–1216. doi: 10.1104/pp.112.205385, PMID: 23296688 PMC3585590

[ref19] Jisha V. Dampanaboina L. Vadassery J. Mithoefer A. Kappara S. Ramanan R. (2015). Overexpression of an AP2/ERF type transcription factor OsEREBP1 confers biotic and abiotic stress tolerance in Rice. PLoS One 10:e0127831. doi: 10.1371/journal.pone.0127831, PMID: 26035591 PMC4452794

[ref20] Lemmon M. A. Schlessinger J. (2010). Cell signaling by receptor tyrosine kinases. Cell 141, 1117–1134. doi: 10.1016/j.cell.2010.06.011, PMID: 20602996 PMC2914105

[ref21] Li X. L. Li J. Q. Lu Y. G. (2007). Research on the construction strategy of rice core collection. J. Shenyang Agri. Univ. 5, 681–687.

[ref600] Li X. Lu Y. Li J. Xu H. Shahid M. Q. (2011). Strategies on Sample Size Determination and Qualitative and Quantitative Traits Integration to Construct Core Collection of Rice (Oryza sativa). Rice Science 18, 46–55. doi: 10.1016/S1672-6308(11)60007-3

[ref22] Li L. H. Lv M. M. Li X. Ye T. Z. He X. Rong S. H.. (2018). The Rice OsDUF810 family: OsDUF810.7 may be involved in the tolerance to salt and drought. Mol. Biol. 52, 567–575. doi: 10.1134/S0026898418040122, PMID: 30113022

[ref23] Li J. Pu L. Han M. Zhu M. Zhang R. Xiang Y. (2014). Soil salinization research in China: advances and prospects. J. Geogr. Sci. 24, 943–960. doi: 10.1007/s11442-014-1130-2

[ref700] Li J. Zhang P. (2012). “Assessment and utilization of the genetic diversity in rice,” in Genetic Diversity in Plants. ed M. Caliskan (London: InTech-Open Access Publisher), 87–102.

[ref24] Lin F. Li S. Wang K. Tian H. Du C. (2020). A Leucine-rich repeat receptor-like kinase, OsSTLK, modulates salt tolerance in rice. Plant Sci. 296:110465. doi: 10.1016/j.plantsci.2020.110465, PMID: 32540023

[ref25] Liu C. Chen K. Zhao X. Wang X. Shen C. Zhu Y.. (2019). Identification of genes for salt tolerance and yield-related traits in rice plants grown hydroponically and under saline field conditions by genome-wide association study. Rice 12:88. doi: 10.1186/s12284-019-0349-z, PMID: 31792643 PMC6889114

[ref800] Livak K. J. Schmittgen T. D. (2002). Analysis of Relative Gene Expression Data using Real-Time Quantitative PCR. Methods 25, 402–408. doi: 10.1006/meth.2001.1262 11846609

[ref26] Lv M. Hou D. Zhang L. Fan J. Li C. Chen W.. (2019). Molecular characterization and function analysis of the rice OsDUF1191 family. Biotechnol. Equipment 33, 1608–1615. doi: 10.1080/13102818.2019.1684843

[ref27] Ma X. Yuan Y. Li C. Wu Q. He Z. Li J.. (2021). Brassinosteroids suppress ethylene-induced fruitlet abscission through LcBZR1/2-mediated transcriptional repression of LcACS1/4 and LcACO2/3 in litchi. Hortic. Res. 8:105. doi: 10.1038/s41438-021-00540-z, PMID: 33931615 PMC8087802

[ref300] Mccouch S. Cho Y. Yano M. Paul E. Blinstrub M. Morishima H.. (1997). Report on QTL nomenclature. Rice Genet Newsl 14., PMID: 26172270

[ref28] Mudgal R. Sandhya S. Chandra N. Srinivasan N. (2015). De-DUFing the DUFs: deciphering distant evolutionary relationships of domains of unknown function using sensitive homology detection methods. Biol. Direct 10:38. doi: 10.1186/s13062-015-0069-2, PMID: 26228684 PMC4520260

[ref29] Mukhopadhyay A. Vij S. Tyagi A. K. (2004). Overexpression of a zinc-finger protein gene from rice confers tolerance to cold, dehydration, and salt stress in transgenic tobacco. Proc. Natl. Acad. Sci. U. S. A. 101, 6309–6314. doi: 10.1073/pnas.0401572101, PMID: 15079051 PMC395965

[ref30] Naveed S. A. Zhang F. Zhang J. Zheng T. Q. Meng L. J. Pang Y. L.. (2018). Identification of QTN and candidate genes for salinity tolerance at the germination and seedling stages in Rice by genome-wide association analyses. Sci. Rep. 8:6505. doi: 10.1038/s41598-018-24946-3, PMID: 29695843 PMC5916932

[ref31] Nayyeripasand L. Garoosi G. A. Ahmadikhah A. (2021). Genome-wide association study (GWAS) to identify salt-tolerance QTLs carrying novel candidate genes in Rice During early vegetative stage. Rice 14:9. doi: 10.1186/s12284-020-00433-0, PMID: 33420909 PMC7797017

[ref32] Ouyang S.-Q. Liu Y.-F. Liu P. Lei G. He S.-J. Ma B.. (2010). Receptor-like kinase OsSIK1 improves drought and salt stress tolerance in rice (*Oryza sativa*) plants. Plant J. 62, 316–329. doi: 10.1111/j.1365-313X.2010.04146.x, PMID: 20128882

[ref33] Park S. Moon J.-C. Park Y. C. Kim J.-H. Kim D. S. Jang C. S. (2014). Molecular dissection of the response of a rice leucine-rich repeat receptor-like kinase (LRR-RLK) gene to abiotic stresses. J. Plant Physiol. 171, 1645–1653. doi: 10.1016/j.jplph.2014.08.002, PMID: 25173451

[ref34] Park G.-G. Park J.-J. Yoon J. Yu S.-N. An G. (2010). A RING finger E3 ligase gene, *Oryza sativa* delayed seed germination 1 (OsDSG1), controls seed germination and stress responses in rice. Plant Mol. Biol. 74, 467–478. doi: 10.1007/s11103-010-9687-3, PMID: 20878348

[ref35] Planas-Riverola A. Gupta A. Betegon-Putze I. Bosch N. Ibanes M. Cano-Delgado A. I. (2019). Brassinosteroid signaling in plant development and adaptation to stress. Development 146:dev.151894. doi: 10.1242/dev.151894, PMID: 30872266 PMC6432667

[ref36] Prasad S. R. Bagali P. G. Hittalmani S. Shashidhar H. E. (2000). Molecular mapping of quantitative trait loci associated with seedling tolerance to salt stress in rice (*Oryza sativa* L.). Curr. Sci. 78, 162–164.

[ref37] Qadir M. Quillerou E. Nangia V. Murtaza G. Singh M. Thomas R. J.. (2014). Economics of salt-induced land degradation and restoration. Nat. Res. Forum 38, 282–295. doi: 10.1111/1477-8947.12054

[ref500] Qi D. L. Han L. Z. Zhang S. Y. (2005). Methods of Characterization and Evaluation of Salt or Alkaline Tolerance in Rice. Journal of Plant Genetic Resources 6, 226–231. PMID: 33420909

[ref38] Quynh-Nga N. Lee Y.-S. Cho L.-H. Jeong H.-J. An G. Jung K.-H. (2015). Genome-wide identification and analysis of *Catharanthus roseus* RLK1-like kinases in rice. Planta 241, 603–613. doi: 10.1007/s00425-014-2203-2 25399351

[ref39] Roy S. J. Tucker E. J. Tester M. (2011). Genetic analysis of abiotic stress tolerance in crops. Curr. Opin. Plant Biol. 14, 232–239. doi: 10.1016/j.pbi.2011.03.002 21478049

[ref40] Ruan S.-L. Ma H.-S. Wang S.-H. Fu Y.-P. Xin Y. Liu W.-Z.. (2011). Proteomic identification of OsCYP2, a rice cyclophilin that confers salt tolerance in rice (*Oryza sativa* L.) seedlings when overexpressed. BMC Plant Biol. 11:34. doi: 10.1186/1471-2229-11-34, PMID: 21324151 PMC3050798

[ref41] Sakamoto H. (2004). Arabidopsis Cys2/His2-type zinc-finger proteins function as transcription repressors under drought, cold, and high-salinity stress conditions. Plant Physiol. 136, 2734–2746. doi: 10.1104/pp.104.046599, PMID: 15333755 PMC523337

[ref42] Schmidt R. Schippers J. H. M. Welker A. Mieulet D. Guiderdoni E. Mueller-Roeber B. (2012). Transcription factor OsHsfC1b regulates salt tolerance and development in *Oryza sativa* ssp japonica. AOB Plants. 2012:pls011. doi: 10.1093/aobpla/pls011 22616023 PMC3357053

[ref43] Shin J.-H. Blay S. Mcneney B. Graham J. (2006). LDheatmap: An R function for graphical display of pairwise linkage disequilibria between single nucleotide polymorphisms. J. Stat. Softw. 16, 1–9. doi: 10.18637/jss.v016.c03

[ref44] Solis C. A. Yong M. T. Vinarao R. Jena K. Chen Z. H. (2020). Back to the wild: on a quest for donors toward salinity tolerant Rice. Front. Plant Sci. 11:323. doi: 10.3389/fpls.2020.00323, PMID: 32265970 PMC7098918

[ref45] Song J. Jinqun L. Jian S. Tao H. Aiting W. Sitong L.. (2018). Genome-wide association mapping for cold tolerance in a Core collection of Rice (*Oryza sativa* L.) landraces by using high-density single nucleotide polymorphism markers from specific-locus amplified fragment sequencing. Front. Plant Sci. 9:875. doi: 10.3389/fpls.2018.00875 30013584 PMC6036282

[ref46] Takano S. Matsu Da S. Unabiki A. F. Furukawa J. I. Yamauchi T. Tokuji Y.. (2015). The rice RCN11 gene encodes β1,2-xylosyltransferase and is required for plant responses to abiotic stresses and phytohormones. Plant Sci. 236, 75–88. doi: 10.1016/j.plantsci.2015.03.022, PMID: 26025522

[ref47] Takehisa H. Shimodate T. Fukuta Y. Ueda T. Yano M. Yamaya T.. (2004). Identification of quantitative trait loci for plant growth of rice in paddy field flooded with salt water. Field Crop Res. 89, 85–95. doi: 10.1016/j.fcr.2004.01.026

[ref48] Tian X. Wang Z. Li X. Lv T. Liu H. (2015). Characterization and functional analysis of Pyrabactin resistance-Like Abscisic acid receptor family in Rice. Rice. 8:28. doi: 10.1186/s12284-015-0061-6, PMID: 26362328 PMC4567572

[ref49] Walia H. Wilson C. Condamine P. Liu X. Ismail A. M. Zeng L.. (2005). Comparative transcriptional profiling of two contrasting rice genotypes under salinity stress during the vegetative growth stage. Plant Physiol. 139, 822–835. doi: 10.1104/pp.105.065961, PMID: 16183841 PMC1255998

[ref50] Wang D. R. Agosto-Pérez F. J. Chebotarov D. Shi Y. Marchini J. Fitzgerald M.. (2018a). An imputation platform to enhance integration of rice genetic resources. Nat. Commun. 9:3519. doi: 10.1038/s41467-018-05538-1, PMID: 30158584 PMC6115364

[ref51] Wang Z. Chen Z. Cheng J. Lai Y. Wang J. Bao Y.. (2012). QTL analysis of Na+ and K+ concentrations in roots and shoots under different levels of NaCl stress in Rice (*Oryza sativa* L.). PLoS One 7:e51202. doi: 10.1371/journal.pone.0051202, PMID: 23236455 PMC3516561

[ref52] Wang R. Jing W. Xiao L. Jin Y. Shen L. Zhang W. (2015). The Rice high-affinity potassium Transporter1;1 is involved in salt tolerance and regulated by an MYB-type transcription factor. Plant Physiol. 168:1076. doi: 10.1104/pp.15.00298, PMID: 25991736 PMC4741328

[ref53] Wang W. Mauleon R. Hu Z. Chebotarov D. Tai S. Wu Z.. (2018b). Genomic variation in 3,010 diverse accessions of Asian cultivated rice. Nature 557:43. doi: 10.1038/s41586-018-0063-9, PMID: 29695866 PMC6784863

[ref54] Wen J.-Q. Oono K. Imai R. (2002). Two novel mitogen-activated protein signaling components, OsMEK1 and OsMAP1, are involved in a moderate low-temperature signaling pathway in rice. Plant Physiol. 129, 1880–1891. doi: 10.1104/pp.006072, PMID: 12177502 PMC166777

[ref55] Wu F. Yang J. Yu D. Xu P. (2020). Identification and validation a major QTL from “sea Rice 86,” seedlings conferred salt tolerance. Agronomy 10:410. doi: 10.3390/agronomy10030410

[ref56] Wu H. Ye H. Yao R. Zhang T. Xiong L. (2015). OsJAZ9 acts as a transcriptional regulator in jasmonate signaling and modulates salt stress tolerance in rice. Plant Sci. 232, 1–12. doi: 10.1016/j.plantsci.2014.12.010, PMID: 25617318

[ref57] Xia K. Ou X. Tang H. Ren W. Ping W. Jia Y.. (2015). Rice microRNA Osa-miR1848 targets the obtusifoliol 14α-demethylase gene OsCYP51G3 and mediates the biosynthesis of phytosterols and brassinosteroids during development and in response to stress. New Phytol. 208, 790–802. doi: 10.1111/nph.13513, PMID: 26083975

[ref1000] Xiaoxue P. Mingyu H. Xiaoying J. Wenqin B. Ling G. Hong W.. (2019). Overexpression of the Thellungiella salsuginea TsIPK2 gene enhances salt tolerance of transgenic rice. J. Plant Nutrit. Fertiliz 25, 741–747. doi: 10.11674/zwyf.18144

[ref58] Xu M.-R. Huang L.-Y. Zhang F. Zhu L.-H. Zhou Y.-L. Li Z.-K. (2013). Genome-wide phylogenetic analysis of stress-activated protein kinase genes in Rice (OsSAPKs) and expression profiling in response to *Xanthomonas oryzae* pv. Oryzicola infection. Plant Mol. Biol. Report. 31, 877–885. doi: 10.1007/s11105-013-0559-2

[ref59] Yang Z. Huang Y. Yang J. Yao S. Zhao K. Wang D.. (2020). Jasmonate signaling enhances RNA silencing and antiviral defense in Rice. Cell Host Microbe 28:89. doi: 10.1016/j.chom.2020.05.001, PMID: 32504578

[ref60] Yang Y. Yan G. (2018). Unraveling salt stress signaling in plants. J. Integr. Plant Biol. 60, 796–804. doi: 10.1111/jipb.12689 29905393

[ref61] Ye Y. Ding Y. Jiang Q. Wang F. Sun J. Zhu C. (2017). The role of receptor-like protein kinases (RLKs) in abiotic stress response in plants. Plant Cell Rep. 36, 235–242. doi: 10.1007/s00299-016-2084-x 27933379

[ref62] Yoshida S. Forno D. A. Cock J. H. Gomez K. A. (1976). *Laboratory Manual for Physiological Studies of Rice.* *3rd Edn.* The International Rice Research Institute, Manila.

[ref63] Yu Z. Duan X. Luo L. Dai S. Xia G. (2020). How plant hormones mediate salt stress responses. Trends Plant Sci. 25, 1117–1130. doi: 10.1016/j.tplants.2020.06.008, PMID: 32675014

[ref64] Yuan J. Wang X. Zhao Y. Khan N. U. Zhao Z. Zhang Y.. (2020). Genetic basis and identification of candidate genes for salt tolerance in rice by GWAS. Sci. Rep. 10:9958. doi: 10.1038/s41598-020-66604-7, PMID: 32561778 PMC7305297

[ref65] Zeng P. Zhu P. Qian L. Qian X. Mi Y. Lin Z.. (2021). Identification and fine mapping of qGR6.2, a novel locus controlling rice seed germination under salt stress. BMC Plant Biol. 21:36. doi: 10.1186/s12870-020-02820-7, PMID: 33422012 PMC7797128

[ref66] Zhang J. Chen K. Pang Y. Naveed S. A. Zhao X. Wang X.. (2017). QTL mapping and candidate gene analysis of ferrous iron and zinc toxicity tolerance at seedling stage in rice by genome-wide association study. BMC Genomics 18:828. doi: 10.1186/s12864-017-4221-5, PMID: 29078746 PMC5658907

[ref900] Zhang P. Li J. Li X. Liu X. Zhao X. Lu Y. (2011). Population Structure and Genetic Diversity in a Rice Core Collection (Oryza sativa L.) Investigated with SSR Markers. PLoS One 6:e27565. doi: 10.1371/journal.pone.0027565 22164211 PMC3229487

[ref67] Zhang Y. Ponce K. Meng L. Chakraborty P. Ye G. (2020). QTL identification for salt tolerance related traits at the seedling stage in indica rice using a multi-parent advanced generation intercross (MAGIC) population. Plant Growth Regul. 92, 365–373. doi: 10.1007/s10725-020-00644-x

[ref68] Zhao X. Dou L. Gong Z. Wang X. Mao T. (2019). BES1 hinders ABSCISIC ACID INSENSITIVE5 and promotes seed germination in Arabidopsis. New Phytol. 221, 908–918. doi: 10.1111/nph.15437 30230549

[ref69] Zhao M. Song J. Wu A. Hu T. Li J. (2018). Mining beneficial genes for aluminum tolerance Within a Core collection of Rice landraces Through genome-wide association mapping With high density SNPs From specific-locus amplified fragment sequencing. Front. Plant Sci. 9:1838. doi: 10.3389/fpls.2018.01838, PMID: 30619409 PMC6305482

[ref70] Zhou Y.-B. Liu C. Tang D.-Y. Yan L. Wang D. Yang Y.-Z.. (2018). The receptor-Like cytoplasmic kinase STRK1 phosphorylates and activates CatC, thereby regulating H2O2 homeostasis and improving salt tolerance in Rice. Plant Cell 30, 1100–1118. doi: 10.1105/tpc.17.01000, PMID: 29581216 PMC6002193

[ref71] Zhuang J. Jiang H.-H. Wang F. Peng R.-H. Yao Q.-H. Xiong A.-S. (2013). A Rice OsAP23, functioning as an AP2/ERF transcription factor, reduces salt tolerance in transgenic Arabidopsis. Plant Mol. Biol. Report. 31, 1336–1345. doi: 10.1007/s11105-013-0610-3

