# Supplementary figures and images for: Mining Beneficial Genes for Salt Tolerance From a Core Collection of Rice Landraces at the Seedling Stage Through Genome-Wide Association Mapping
Source: Front Plant Sci. 2022 Apr 26;13:847863. doi: 10.3389/fpls.2022.847863 (PMC9087808; doi:10.3389/fpls.2022.847863)

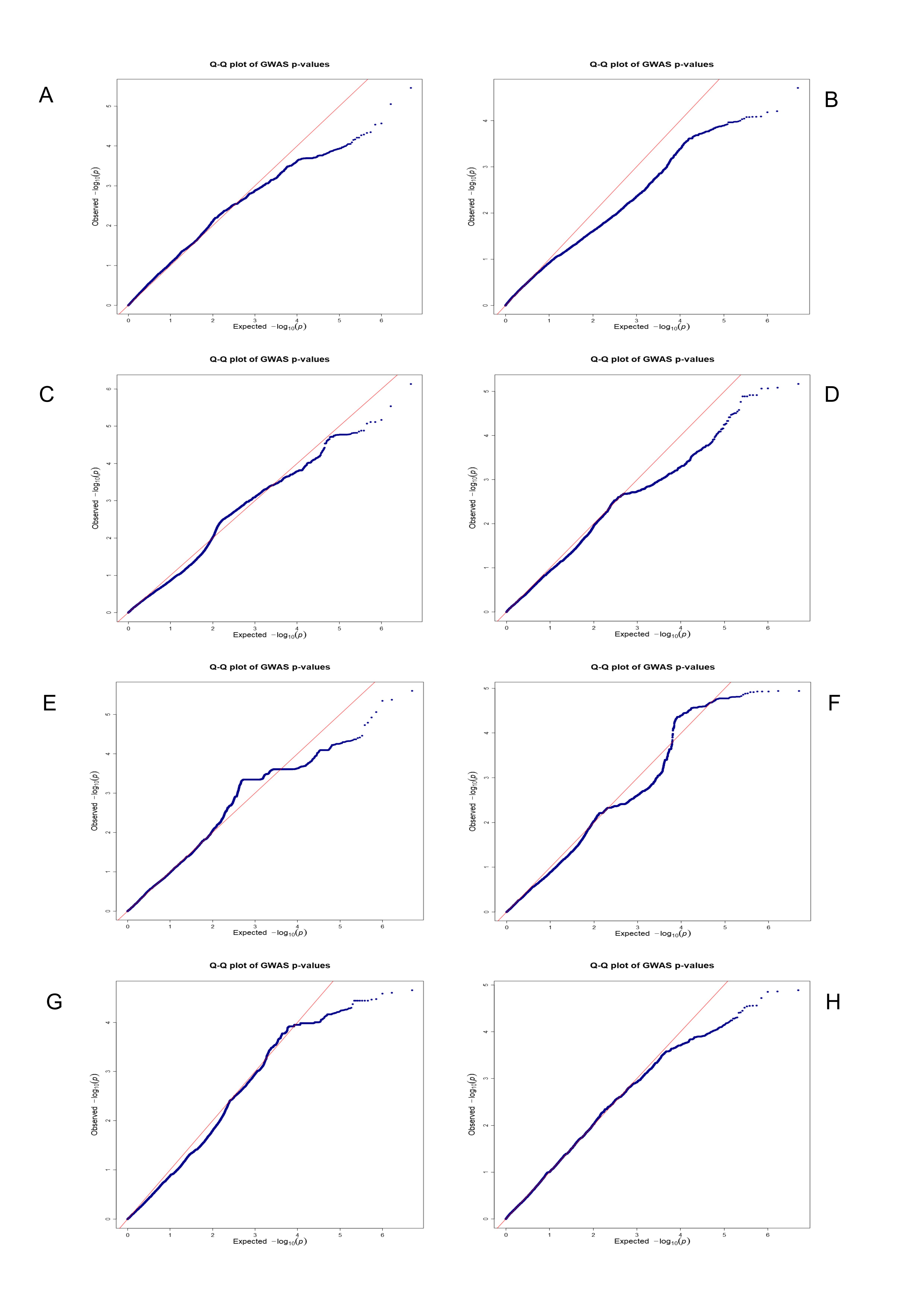

Supplement: Supplementary Figure S1 — QQ plots of genome-wide association studies for the eight traits related to ST. A-H: QQ plots for RNC, SKC, SNC, RN/K, RTRL, RTRSA, RTRV, and RSN/K. [file Image_1.JPEG]

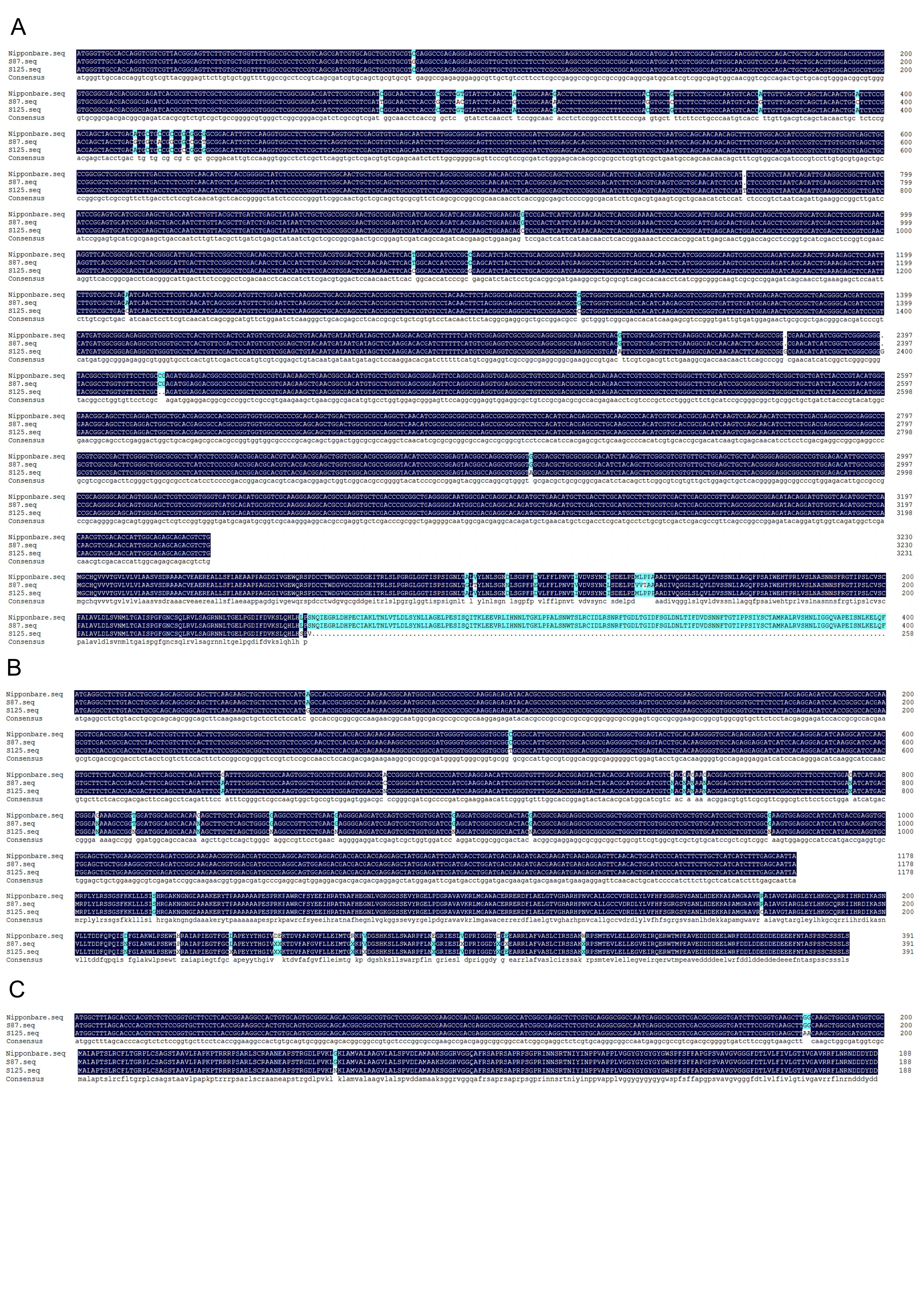

Supplement: Supplementary Figure S2 — (A–C) DNA sequence analysis and amino acid sequence analysis for three candidate genes. [file Image_2.JPEG]

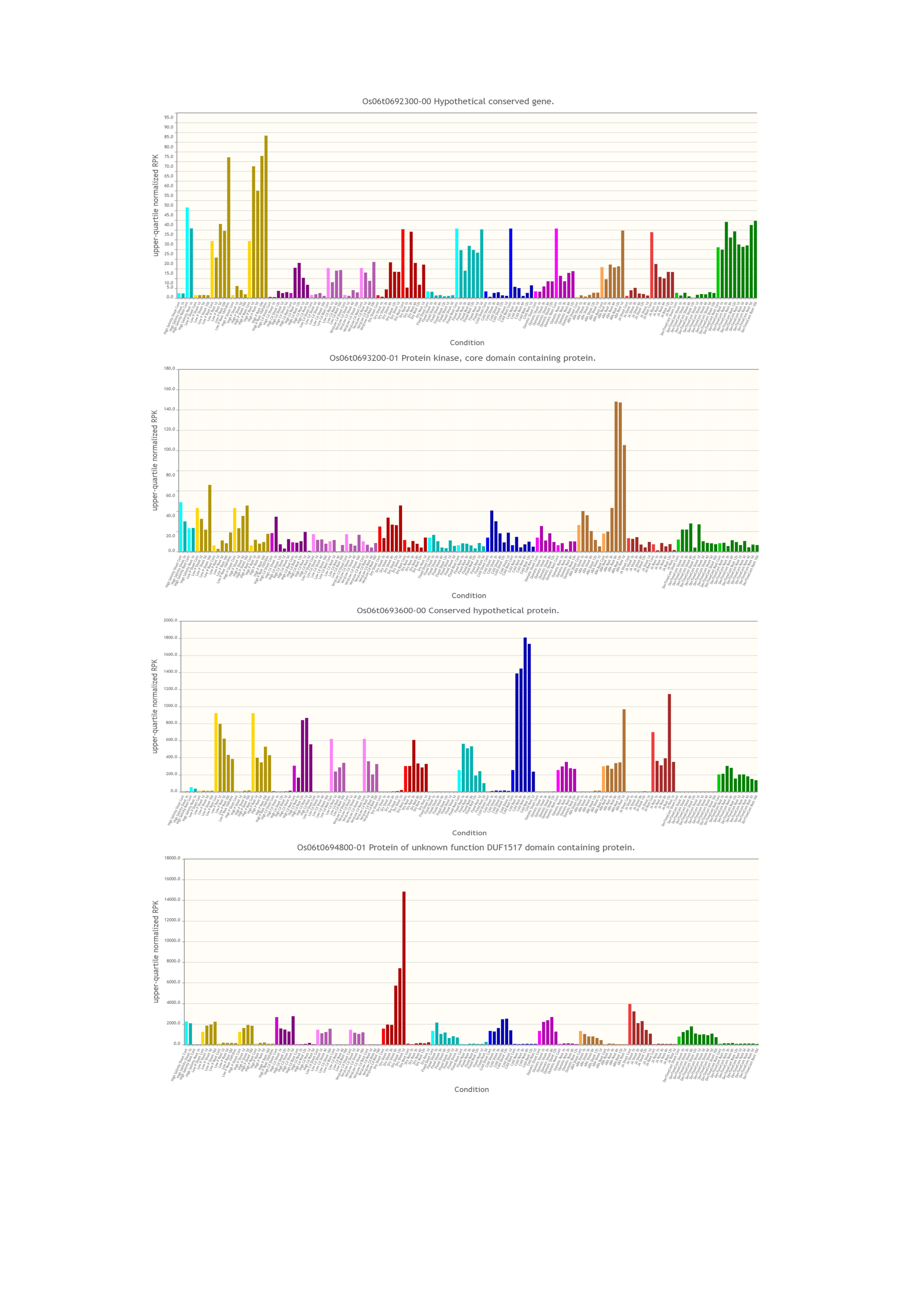

Supplement: Supplementary Figure S3 — Expression profiles in rice seedling under the various environmental conditions. [file Image_3.JPEG]
